# Supplementary material for: North Atlantic temperature control on deoxygenation in the northern tropical Pacific
Source: Nat Commun. 2024 Sep 10;15:7919. doi: 10.1038/s41467-024-52197-6 (PMC11387393; doi:10.1038/s41467-024-52197-6)
Supplement: Supplementary file 1 — Supplementary Information [file 41467_2024_52197_MOESM1_ESM.pdf]

Supplementary Information

Supplementary note 1: Varve-based versus carbon dating-based sedimentation rates in the laminated sequences

Core MD02-2515 was collected near the locality of a piston core with a published record of varved (annually laminated) Holocene and Deglacial sediments (JPC56, 27°28.16"N 112°06.26'W, water depth 818 m;(*1, 2*)). The varves consists of an alternation between diatom rich and clay/silt rich sediment, which reflect the seasonal alternation between high productivity during the late fall/spring upwelling season and increased lithogenic fluxes during summer (*1, 3*). MD02-2515 recovered 73m of sediment in total, comprising Holocene/Deglacial sediments similar to JPC56 as well as a more intermittently laminated/varved sequence of glacial sediments.

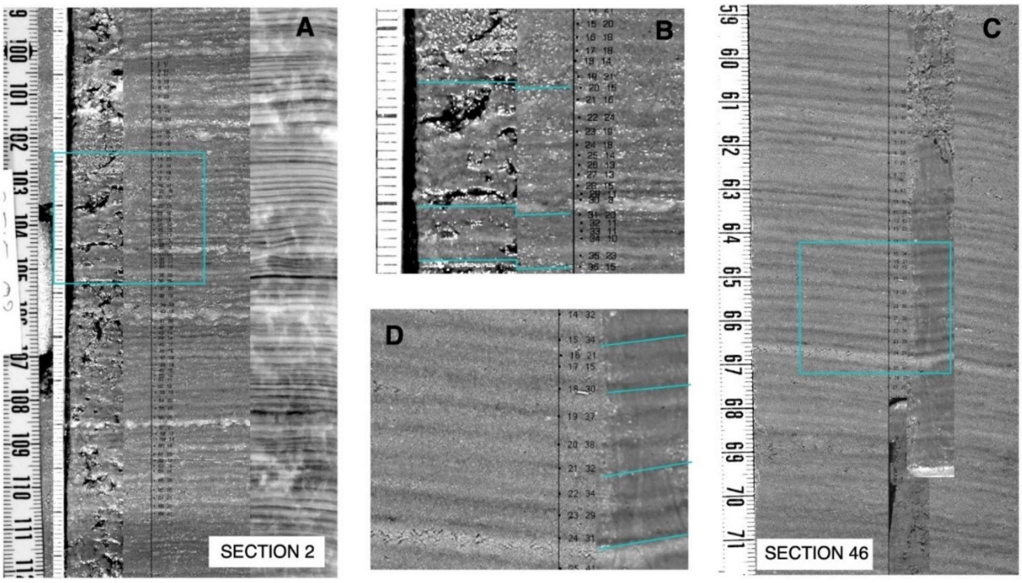

Supplementary Figure 1: Examples of digital core photographs combined with slab sample photographs and X-ray image as used to transfer varve counts onto the sediment slabs for annual resolution sampling. **A**. core photograph of section 2 with line along which varves were measured and counted, with **B** showing enlargement of marked area. The numbers along the black line represent varve number and thickness in pixels respectively; blue lines indicate how individual varves are traced onto the slab sample. **C** and **D**. Core photograph of section 46, with black markings and blue lines as in A and B.

The overall age framework for MD02-2515 has been established using radiocarbon analyses on organic matter (*4*). Annual-resolution floating age models were constructed for distinctly laminated intervals from varve-counts on digital core photographs. Varve thickness and stratigraphic position were measured on a computer screen using public-domain image analysis software in combination with a macro that translates subsequent mouse-clicks on varve- boundaries into position and thickness (Supplementary Figure 1). Data for individual images are imported into a spreadsheet and stitched together into a continuous record. For the Holocene and Deglacial sediments, which are virtually continuously laminated, there is a good match between the ^{14}C ages and the varve-based age model (Supplementary Figure 2). For the glacial part of the core, floating varve-based age models for discrete laminated intervals are tied to an absolute age using the radiocarbon dates.

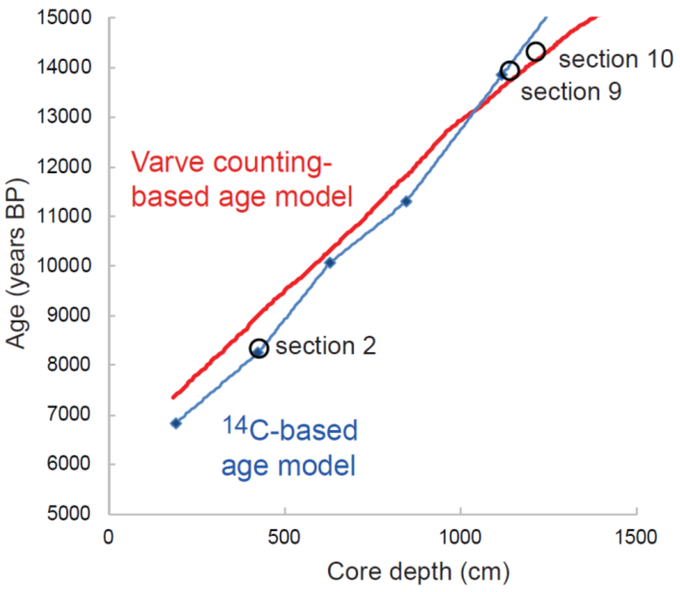

Supplementary Figure 2: Cross plot of core depth against age for both the radiocarbon and varve-based age models for the Holocene and Deglacial part of MD02-2515. The similarity of the two age models illustrates that the sediment provides a virtually complete record at annual resolution without major gaps.

Supplementary note 2: SSTs, export fluxes and Decadal and sub-decadal Pacific climate oscillations

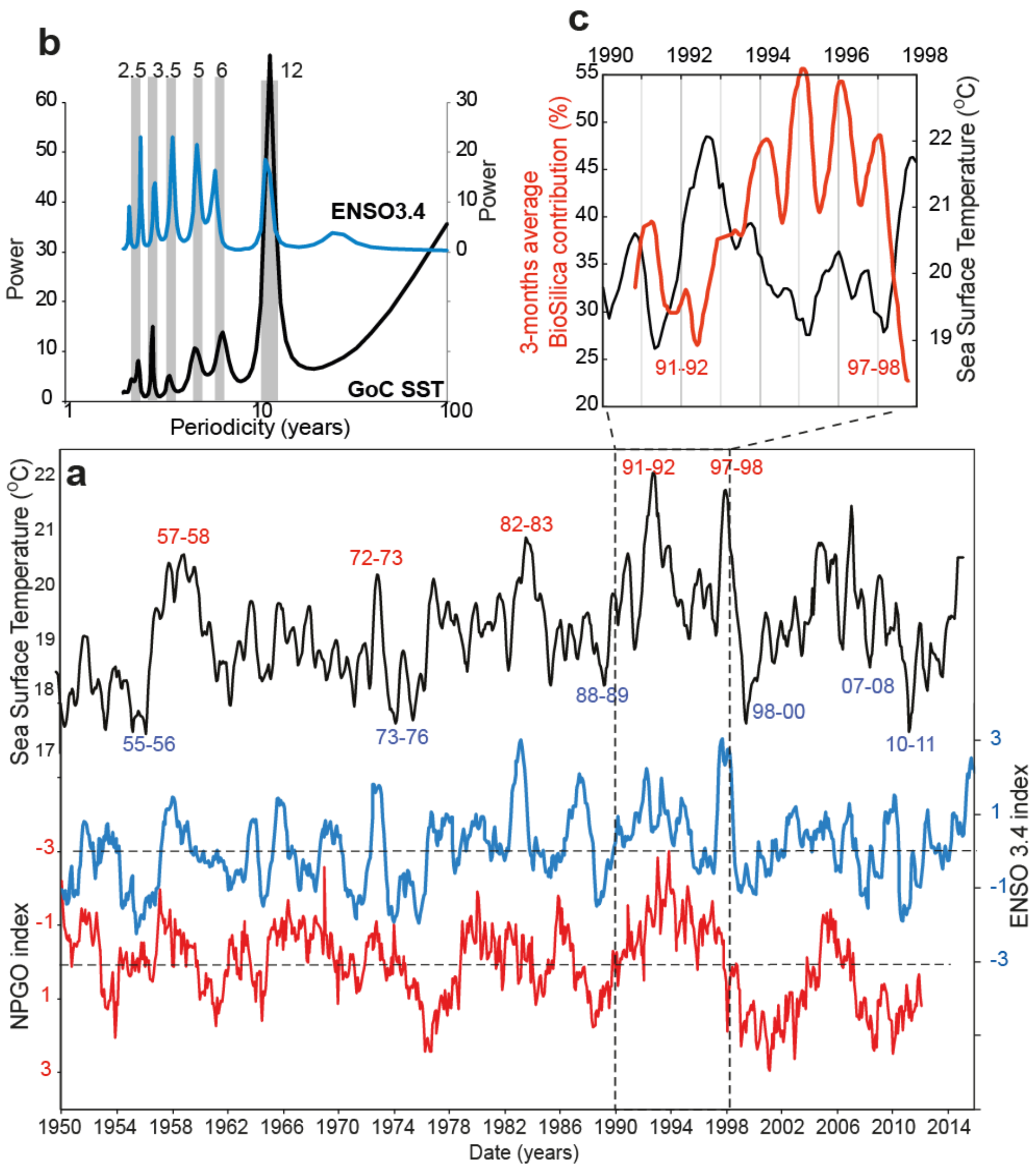

Supplementary Figure 3:

(a) NPGO and ENSO 3.4 indices (http://research.jisao.washington.edu) and sea surface temperatures in the Gulf of California (GoC) for the period 1950-2015 (https://icoads.noaa.gov/) (*5*). (b) Time series analyses in the frequency domain using spectral analyses with various windowing settings (Blackman-Tuckey -not shown- and Max Entropy) for the ENSO3.4 index and the SST record. (c) Temporal changes in the SST record of the GoC (black) and biogenic silica export fluxes to the sediment over the period 1990-1998 from Thunell at al. (*3*).

Cold SSTs excursions in the GoC correspond to active upwelling periods whilst warm SSTs are symptomatic of surface stratification and tropical surface water invading the GoC (Supplementary Figure 3a, ref(*6*)). These excursions are in phase with the ENSO 3.4 index and the NPGO index, a climate fluctuation reflected by changes in the intensity of the central and eastern branches of the North Pacific gyre circulations and the properties of the California Current(*7*). Time series analyses in the frequency domain using spectral analyses with various windowing settings (Blackman-Tuckey -not shown- and Max Entropy) show a good match between the ENSO3.4 index and the SST record spectra, with corresponding periodicities and power at sub-decadal scale (Supplementary Figure 3b). The SST spectrum shows an additional peak at about 12-year periodicity that corresponds to the cyclicity of the NPGO climate variability.

Temporal changes in upwelling dynamics reflected in the GoC SST record control the biogenic and detrital export fluxes to the sediment. In Supplementary Figure 3c, we show 3 month-average contributions (% of total weight accumulation per varve couplets) of biogenic opal (diatoms) calculated from sediment trap data (*3, 6*). During El Niño events such as in 1991-92, ocean stratification and wet climate on land drive marine productivity down and increases detrital (riverine) inputs to the sea floor causing the relative annual biogenic silica contribution to be low (20%). This pattern is repeated during the 1998 El Nino event although the sequence is incomplete. During normal years, upwelling of cold and nutrient subsurface waters promotes marine productivity and dryness on land resulting in high biogenic silica export and decreased dilution of the biogenic fluxes by riverine inputs (Supplementary Figure 3c). This results in high relative annual biogenic silica contribution. We reconstruct annual biogenic silica contribution in our varved sections as an indicator of upwelling related biological production.

Supplementary note 3: Spectral analyses and cross-correlations

Annually resolved Nitrogen isotopes records in 10 sections of our Guaymas basin sediment core were analysed in the frequency domain using spectral analyses with various windowing settings (Blackman-Tuckey -not shown- and Max Entropy, see method section). The main recurring periodicities were identified in the 10 records, namely around 80, 35, 25, 10 and 5-8 years. These periodicities are similar to today's AMO, PDO, NPGO and ENSO observed periodicities (*8-10*). The data in table S1 show a predominance of the multidecadal periodicities compared to the shorter periodicities in all records and the absence of clear AMO-like periodicities in the glacial and stadial sections #26 and #17, respectively.

**Relative power in unfiltered records**										
**Period**	***Section III***	***Section 2***	***Section 9***	***Section 10***	***Section 17***	***Section 26***	***Section 45***	***Section 46***	***Section 47***	***Sectio n 50***
AMO (80 years)	27.5	43.3	35.2	13.8	not found	not found	5.5	53.9	26.9	34.0
AMO (30-40 years)	14.3	18.4	11.0	16.2	not found	not found	25.1	11.4	29.7	22.7
PDO (25 years)	33.7	10.2	31.0	36.7	61.3	54.2	50.0	10.0	27.4	25.2
NPGO (10 years)	13.9	10.1	10.7	26.0	18.5	25.7	11.9	16.6	10.3	8.1
ENSO (5-8 years)	10.6	18.1	12.1	7.3	20.2	20.1	7.6	8.1	5.7	10.0

Supplementary Table 1: Relative power (in %) of the various periodicities identified in each denitrification ($\delta^{15}N$) record.

We examined whether there was significant and consistent relationship between the upwelling/biological productivity indicator (Biogenic Silica) and the denitrification indicator ($\delta^{15}N$) in the 10 selected records. Supplementary Figure 4 shows the cross-correlation plots for each couple of annually resolved records (red line compared to the cross-correlation plot of identical records in black, for reference). Section 10, 45, 46 show some degree of correlation, albeit not statistically significant between Biogenic silica and denitrification, with a correlation coefficient of around 0.4 at 0 year lag, while the other 7 records show no positive correlation or even slightly negative relationship between $\delta^{15}N$ and biogenic silica. Detrended records, i.e. filtered to remove variability at periodicity greater than 75 years, show even lower degrees of correlation than the non-detrended records which are shown in Supplementary Figure 4. This indicate that at multi- to sub-decadal timescales, there is no correlation between

biogenic export and hypoxia-induced denitrification. There is no systematic lead-lag relationship between the 2 parameters. Coherence spectra between biogenic silica and nitrogen isotope time series analyses are shown in Supplementary Figure 5 and corroborate our findings that there is no significant relationship between productivity and denitrification changes in the GoC. The spectra are below the 95% confidence limit for most of the frequencies and there are no frequencies at which the spectra show systematically good coherence across all sections.

The cross-correlation plot for Section 26 (non-detrended) stands out and exhibits a negative correlation between Biogenic silica and $\delta^{15}N$ suggesting that denitrification decreases with increasing biogenic export. It is worth noting that this negative correlation is not seen between the detrended records (Supplementary Figure 4). Section 26 was deposited just before the last glacial maximum (26 ka BP). It has been shown that denitrification intensity was dramatically reduced during the last glacial maximum in the ETNP (*11-13*) and therefore, it is not excluded that the nitrogen isotopic signal recorded in this section is only partly influenced by denitrification (in response to hypoxia) and responded to other biogeochemical processes such as nitrate utilisation by the biota(*14*). Under strong upwelling conditions and in absence of, or reduced hypoxia/denitrification, biological productivity and $\delta^{15}N$ are expected to be negatively correlated. Therefore, we do not include section 26 in for the spectral analyses of multi- to sub-decadal deoxygenation changes (section 1 of the paper).

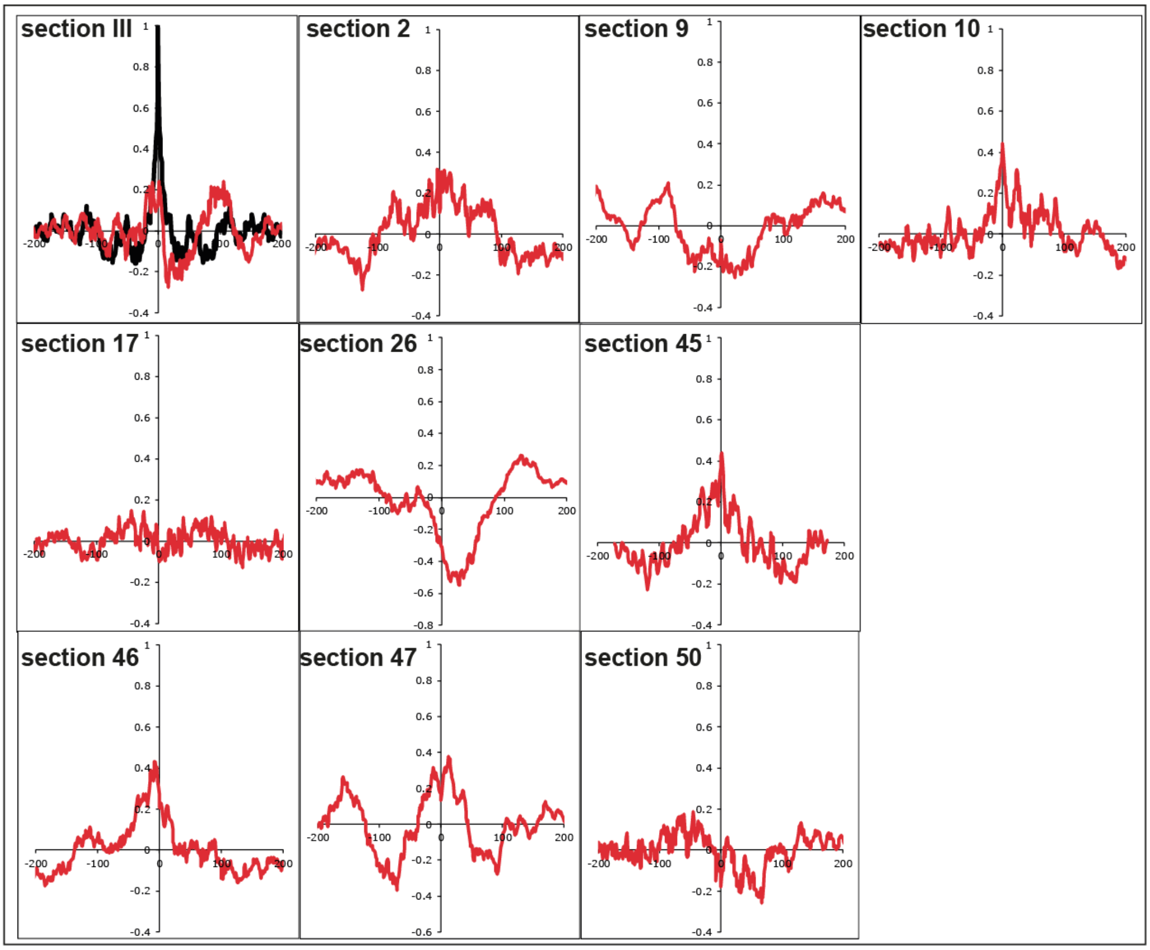

Supplementary Figure 4: Cross correlation between nitrogen isotope and biogenic silica records (red). Example of cross-correlation plot between identical records is shown in black.

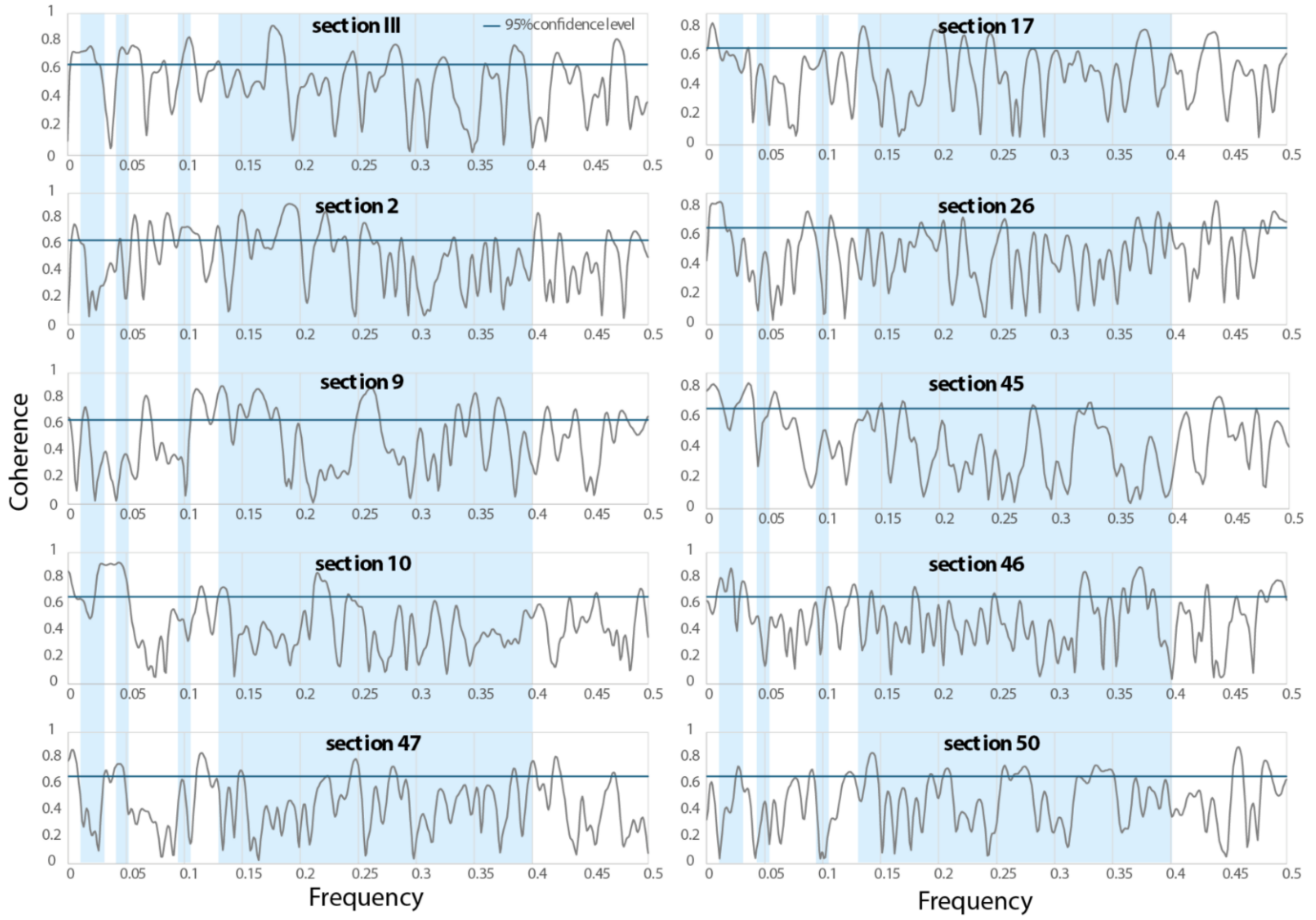

Supplementary Figure 5: Coherence spectra (BTukey using a Bartlett window) between biogenic silica and Nitrogen isotope time series analyse for each section. Non-zero coherence is higher than 0.664122. The error estimation on the power spectrum is 0.415243 < ∆Power / Power < 4.8491. The 95% (confidence levels are shown as a blue line). Blue boxes correspond to the frequency ranges associated with the AMO, PDO, NPGO and ENSO.

Supplementary note 4: Current velocity data re-analyses

We plot the depth profile of annual mean velocity (SODA- Simple Ocean Data Assimilation- data) versus longitude and latitude showing the location of the core of the Equatorial Under Current (EUC) around 100 m water depth. This allow us to identify the space domain considered for the EUC maximum velocity time series analyses presented in Figures 5 and 7 of the manuscript as well as EUC mean climatology (Figure 4 C and D), EUC mean volume transport (Supplementary Figure 8) and velocity anomalies (Figure 4 and Supplementary Figure 9).

We calculate Δu as the difference in EUC maximum zonal velocity between the western (longitude: 140°E-170°W) and eastern (170°W-100°W) Equatorial Pacific between 1880-2012 (Supplementary Figure 7). The velocity gradient Δu increases (decreases) during AMO- (AMO+), indicating a strengthening (reduction) of the eastward acceleration of the EUC in tandem with shifts in the AMO.

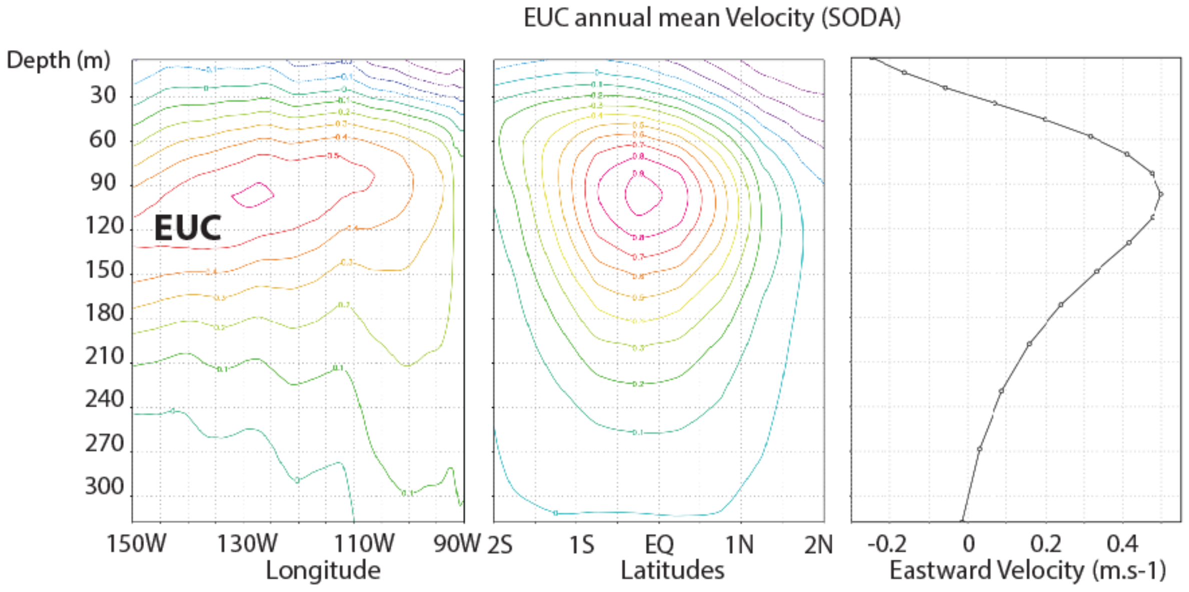

Supplementary Figure 6: From left to right, vertical cross-section of the annual-mean climatological zonal velocity (m.s^{-1}) averaged between 2°S-2°N across the Pacific, vertical cross-section of the annual-mean climatological zonal velocity (m s^{-1}) averaged between 210°-270°E around the equator and the vertical profile of the annual-mean climatological zonal velocity (m s^{-1}) averaged over (210°-270°E; 2°S-2°N). In all the panels, the y axis represents depth (m) below the surface.

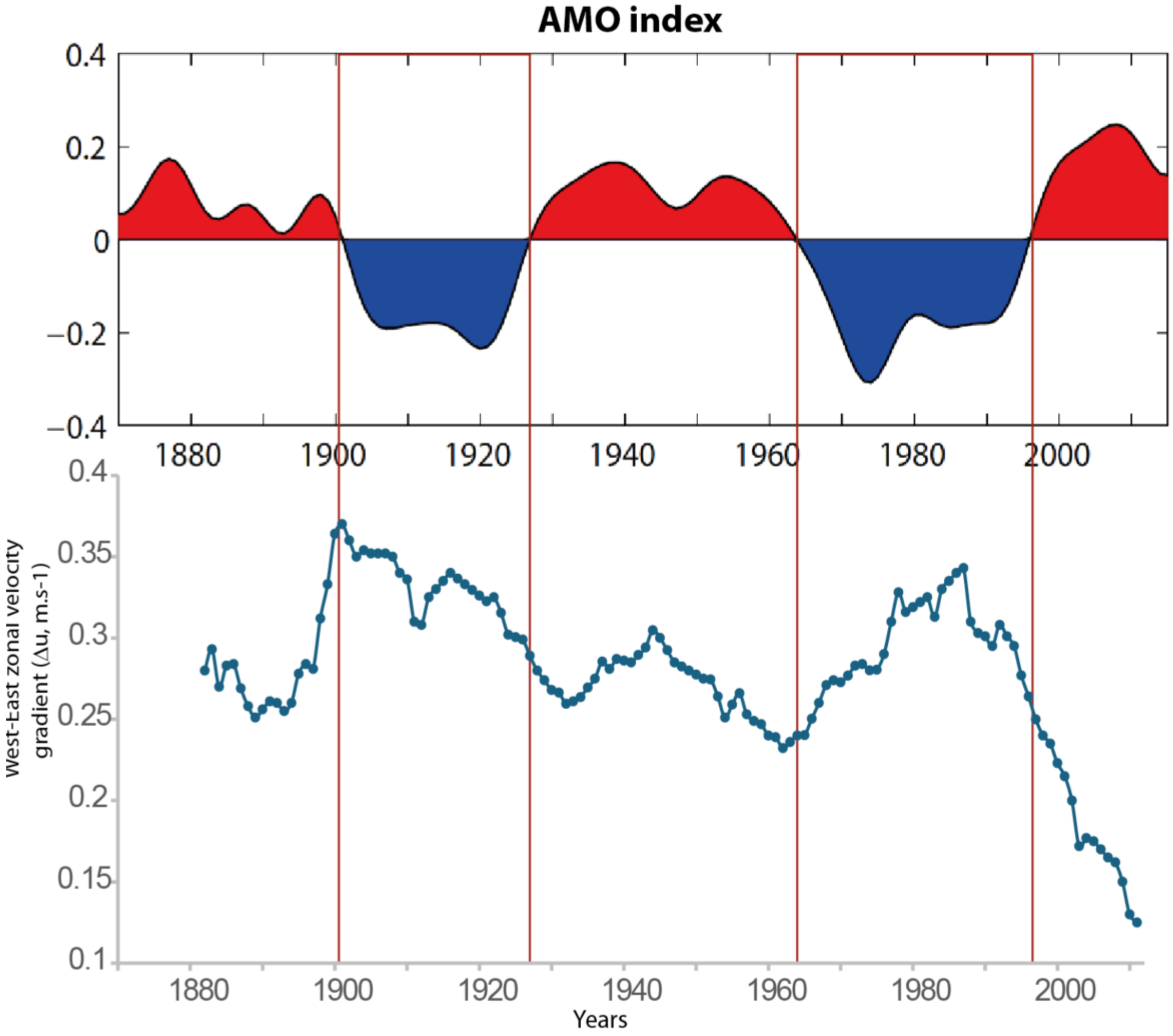

Supplementary Figure 7: Gradient in Equatorial Under Current (EUC) maximum velocity (Δu) between the western (120°E-150°W) and eastern (150°-100°W) Equatorial Pacific compared with the AMO index over the period 1880-2012.

We estimate the mean volume transport variation over the period 1880-2012. We define EUC transport as the mean eastward velocity integrated over ±2.5◦ latitude around the Equator and between 0–350 m, similarly to previous studies (*15, 16*). The results are plotted below in Sv. The transport derived from the mean velocity shows a similar trend to the EUC max velocity. The EUC volume transport increases during periods of AMO- by around 40% during the AMO- of 1900-1930 and roughly doubles during the AMO- period of 1960-1995 compared to AMO+ periods.

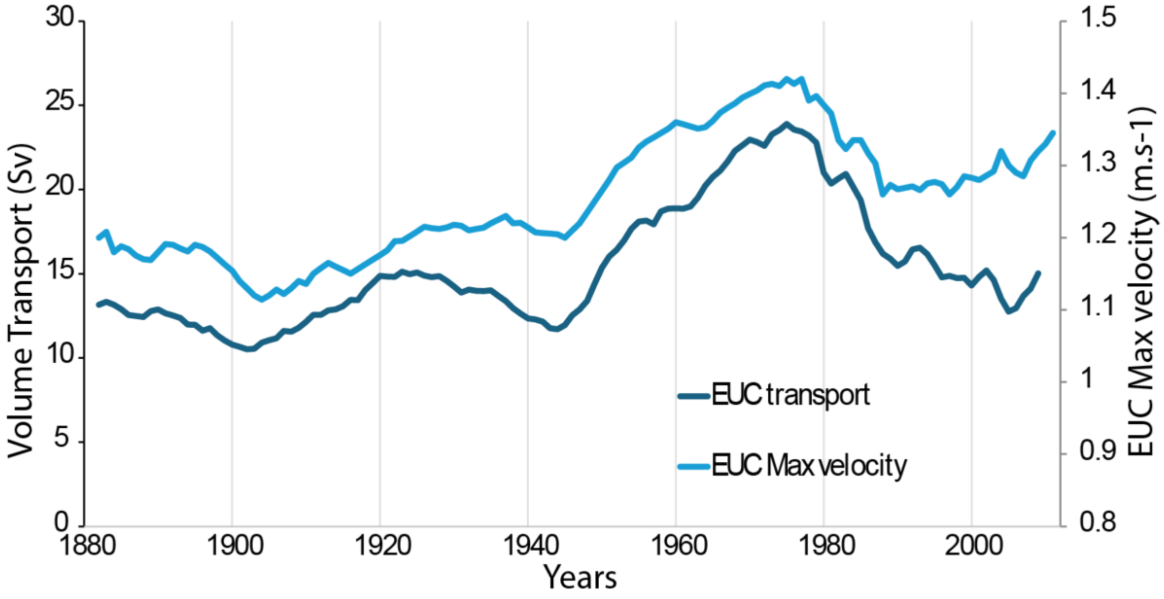

Supplementary Figure 8: Equatorial Under Current (EUC) maximum velocity (m.s-[1]) and volume transport (Sv) between 150°-100°W during the period1880-2012.

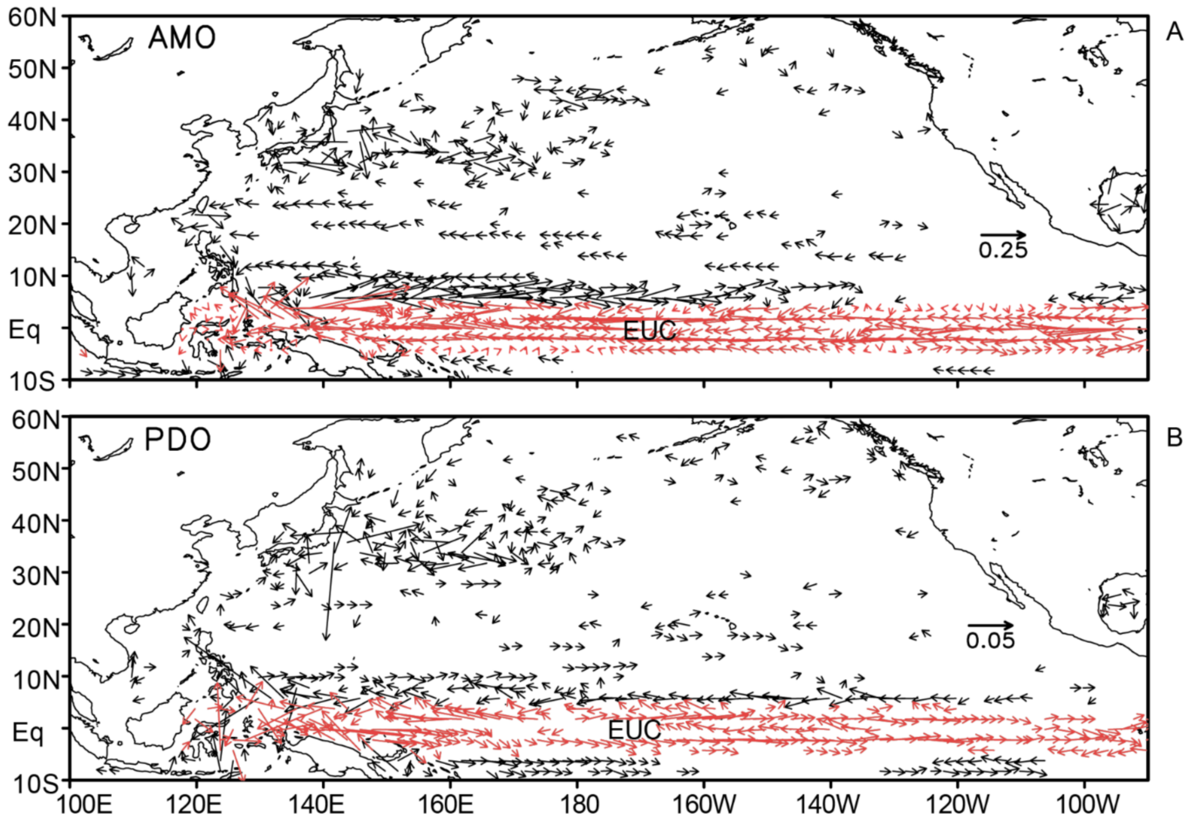

Supplementary Figure 9**:** Regressions of observed low-pass filtered annual-mean ocean currents (m s-1) at 96 m below the surface on the (A) positive AMO and (B) positive PDO indices shown in Figure 1 during 1950-2008. All data were linearly detrended prior to the analysis. Oceanic current data are from the Simple Ocean Data Assimilation (SODA) dataset version v2p2p4 at 0.5° × 0.5° resolution. The red arrows highlight the currents within the 5°S-5°N range.

Supplementary note 5: Annually resolved denitrification records.

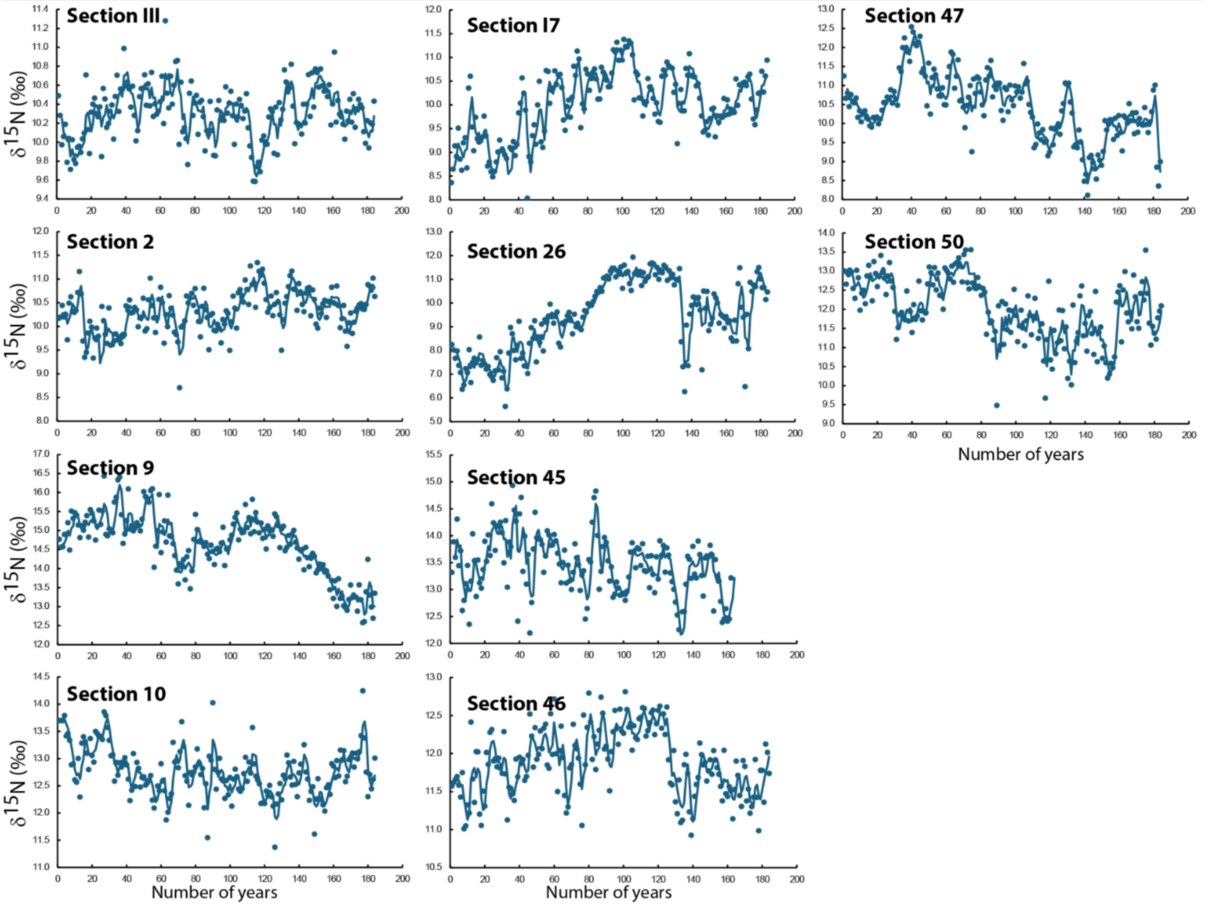

Supplementary Figure 10: Annually resolved (circles) and 3-year averaged (solid line), 200 year-long $\delta^{15}N$ (‰) record for all sections studied showing interannual to multidecadal variability in deoxygenation.

Supplementary Information References

1. J. Pike, A. E. S. Kemp, Records of seasonal flux in Holocene laminated sediments, Gulf of California. *Geological Society, London, Special Publications* **116**, 157-169 (1996).
2. L. D. Keigwin, G. A. Jones, Deglacial climatic oscillations in the Gulf of California. *Paleoceanography* **5**, 1009-1023 (1990).
3. R. C. Thunell, Seasonal and annual variability in particle fluxes in the Gulf of California: A response to climate forcing. *Deep Sea Research I* **45**, 2059-2083 (1998).
4. L. Pichevin *et al.*, Silicic acid biogeochemistry in the Gulf of California: Insights from sedimentary Si isotopes. *Paleoceanography* **27**, (2012).
5. M. Robinson, Atlas of monthly mean sea surface and subsurface temperatures in the Gulf of California. *San Diego Society of Natural History Memoirs* **5**, 1–97 (1973).
6. C. Sancetta, Diatoms in the Gulf of California - Seasonal Flux Patterns and the Sediment Record for the Last 15,000 Years. *Paleoceanography* **10**, 67-84 (1995).
7. E. Di Lorenzo *et al.*, North Pacific Gyre Oscillation links ocean climate and ecosystem change. *Geophysical Research Letters* **35**, (2008).

8. K. B. Ólafsdóttir, Á. Geirsdóttir, G. H. Miller, D. J. Larsen, Evolution of NAO and AMO strength and cyclicity derived from a 3-ka varve-thickness record from Iceland. *Quaternary Science Reviews* **69**, 142-154 (2013).
9. S. Minobe, Resonance in bidecadal and pentadecadal climate oscillations over the North Pacific: Role in climatic regime shifts. *Geophysical Research Letters* **26**, 855-858 (1999).
10. E. Di Lorenzo *et al.*, Central Pacific El Niño and decadal climate change in the North Pacific Ocean. *Nature Geoscience* **3**, 762-765 (2010).
11. C. Pride *et al.*, Nitrogen isotopic variations in the Gulf of California since the last deglaciation: Response to global climate change. *Paleoceanography* **14**, 397-409 (1999).
12. L. E. Pichevin *et al.*, Interhemispheric leakage of isotopically heavy nitrate in the eastern tropical Pacific during the last glacial period. *Paleoceanography* **25**, (2010).
13. R. S. Ganeshram, T. F. Pedersen, S. E. Calvert, G. W. McNeill, M. R. Fontugne, Glacial-interglacial variability in denitrification in the world's oceans: Causes and consequences. *Paleoceanography* **15**, 361-376 (2000).
14. M. Altabet, and R. Francois, *The use of nitrogen isotopic ratio for reconstruction of past changes in surface ocean nutrient utilization*. M. K. R. Zahn, L. Labeyrie, and T. E Pederson Ed., Carbon cycling in the glacial ocean: Constraints on the ocean's role in global change (Springer-Verlag p. 281-306, 1994).
15. A. Stellema, A. Sen Gupta, A. Taschetto, M. Feng, Pacific Equatorial Undercurrent: Mean state, sources, and future changes across models. *Frontiers in Climate* **4**, (2022).
16. K. B. Karnauskas, G. C. Johnson, R. Murtugudde, An Equatorial Ocean Bottleneck in Global Climate Models. *Journal of Climate* **25**, 343-349 (2012).
